# Clinical Outcomes and Cost-effectiveness between the Sentire® and da Vinci® systems in Robot-assisted Radical Prostatectomy

**DOI:** 10.1590/S1677-5538.IBJU.2024.0706

**Published:** 2025-03-25

**Authors:** Run-da Jiao, Zheng Wang, Xian-gui Kong, Shou-yan Tang, Dan Xia, Zhen-jie Wu, Jian-chao Liu, Li-hua Liu

**Affiliations:** 1 Chinese PLA General Hospital Department of Medical Innovation and Research Beijing China Department of Medical Innovation and Research, Chinese PLA General Hospital, Beijing 100853, China; 2 Chinese PLA General Hospital Graduate School Beijing China Graduate School, Chinese PLA General Hospital, Beijing 100853, China; 3 Naval Medical University Changhai Hospital Department of Urology Shanghai China Department of Urology, Changhai Hospital, Naval Medical University, Shanghai 200433, China; 4 The First Affiliated Hospital of Zhejiang University School of Medicine Department Urology Hangzhou China Department Urology, The First Affiliated Hospital of Zhejiang University School of Medicine, Hangzhou 310009, China

**Keywords:** Robotic Surgical Procedures, Prostatic Neoplasms, Cost-Effectiveness Analysis

## Abstract

**Objective:**

Robotic surgery has enhanced minimally invasive procedures with greater precision and control, but high costs have limited its widespread adoption. The Sentire® surgical system is hypothesized to achieve clinical outcomes comparable to those of the da Vinci® system while demonstrating superior cost-effectiveness in robot-assisted radical prostatectomy (RARP) procedures. This study aimed to compare RARP outcomes using Sentire® and da Vinci®, focusing on clinical efficacy and economic impact.

**Materials and Methods:**

A retrospective analysis was conducted at three high-volume urology centers in China, including 22 patients who underwent RARP with the Sentire® system and 287 patients who underwent RARP with the da Vinci® system. After 1:3 propensity score matching (PSM), 66 patients were successfully matched in the control group. Perioperative outcomes and cost metrics were assessed. Key measures included operative and console times, docking time, blood loss, recovery, positive surgical margins, surgeon evaluations of performance and comfort, and cost-effectiveness.

**Results:**

The Sentire® group had a longer median operative time (143 vs. 112 minutes, p=0.024), while console time (85 vs. 76 minutes, p=0.323) and docking time (9.0 vs. 6.0 minutes, p=0.279) were comparable. Blood loss was also similar between the groups (p=0.093). Positive surgical margin rates were 22.7% for Sentire® and 20.0% for da Vinci® (p=1.000), and no significant differences were observed in pathological ISUP grades or prostate volumes (p=0.327 and p=0.856, respectively). At 1-year follow-up, PSA recurrence was observed in 3 patients in the Sentire® group (4.5%) and 4 in the control group (6.1%) (p=0.625), with similar median PSA levels (0.012 vs. 0.014 ng/mL, p=0.410). Urinary continence rates were also comparable at 1, 3, and 12 months (all p > 0.05). Cost-effectiveness analysis revealed lower total and direct costs in the Sentire® group, including surgery expenses ($8,750 vs. $10,500, p=0.021), although differences in consumable and indirect costs were not statistically significant. Surgeon satisfaction scores for performance and comfort were slightly better for Sentire® but did not reach statistical significance (p > 0.05).

**Conclusion:**

This study demonstrates that the Sentire® system is well-suited for urological surgeries, offering comparable clinical outcomes and shorter hospital stays while improving cost-effectiveness compared to the da Vinci® system.

## INTRODUCTION

Robotic surgery represents a significant advancement in minimally invasive procedures, characterized by enhanced precision, flexibility, and control surpassing human capabilities (1). This technology provides surgeons with high-definition, three-dimensional visualization and exceptional accuracy in manipulating robotic arms, making complex surgeries less invasive. Robotic systems extend the surgeon's ability to perform delicate operations with increased steadiness and reduced fatigue, leading to improved patient outcomes, shorter recovery times, and fewer complications. Today, the landscape of surgical robotics is rapidly expanding with the introduction of various systems designed to meet diverse surgical needs.

As robotic systems continue to evolve, clinical applications have also seen ongoing advancements. Among these, robot-assisted radical prostatectomy (RARP) stands out as a representative procedure and has become the standard treatment for clinically localized prostate cancer (2). The procedure's requirements for operating in confined pelvic spaces, the critical need for precise dissection and suturing, the necessity to preserve neurovascular bundles for sexual function and urinary continence, and the complex reconstructive demands ideally demonstrate the capabilities of robotic systems, making RARP a perfect exemplar of how robotic technology can enhance surgical precision and patient outcomes (3–5). Consequently, many novel robotic systems have adopted RARP as a benchmark procedure to demonstrate the effectiveness and safety of new robotic technologies during their early clinical applications (6–8).

Despite the advantages of robotic surgery, it is often perceived to be more costly compared to traditional open or laparoscopic surgeries, especially when a new technology is first introduced, leading to even higher expenses (9–11). The substantial upfront investment required for robotic systems, along with the ongoing costs of maintenance and specialized instruments, have been a significant barrier to their widespread adoption (12). Previous studies have primarily focused on the clinical efficacy and safety of robotic-assisted procedures, there has been comparatively less emphasis on the cost-effectiveness and health economics of these technologies.

The Sentire® C1000 (hereinafter referred to as Sentire®), a novel multi-arm surgical robot independently developed by Cornerstone Technology (Shenzhen) Limited, has demonstrated stable performance and robust safety through extensive preliminary animal experiments. Specifically designed to balance clinical efficacy with cost-effectiveness, Sentire® represents a significant advancement in providing high-quality yet more affordable options for robotic-assisted surgery. This study aims to evaluate whether the Sentire® surgical system achieves clinical outcomes comparable to those of the da Vinci® surgical system, while simultaneously assessing its potential advantages in cost-effectiveness. By employing the propensity score matching (PSM) method to minimize potential biases, this investigation conducts a comprehensive comparison between the two systems, focusing on both clinical efficacy and economic evaluation, with particular emphasis on assessing Sentire®'s performance.

## MATERIALS AND METHODS

### Study Design and Participants

This retrospective study collected data from 22 prostate cancer patients who underwent RARP using the Sentire® robotic system between August 2023 and December 2023 across three high-volume urology centers in China. This study received ethical approval from all participating hospitals: in our institutions (Approval No. 2023-002-01), (Approval No. CHEC2023-117) and (PRO20210241). Additionally, 287 RARP patients treated with the da Vinci® robotic system during the same period were identified. Following 1:3 propensity score matching (PSM), 66 patients from the da Vinci® group were successfully matched as the control group for comparison.

In this retrospective study, data were collected from medical records of patients who underwent RARP with the Sentire® or da Vinci® robotic systems. Inclusion criteria were patients aged 18 to 80 years, a Body Mass Index (BMI) between 18 and 30 kg/m^2^, and a confirmed diagnosis of prostate cancer by biopsy. Patients with distant metastasis, severe comorbidities, a history of prior urological surgery or malignancy, or incomplete medical records were excluded from the analysis.

### Systems and Interventions

**Figure 1:**
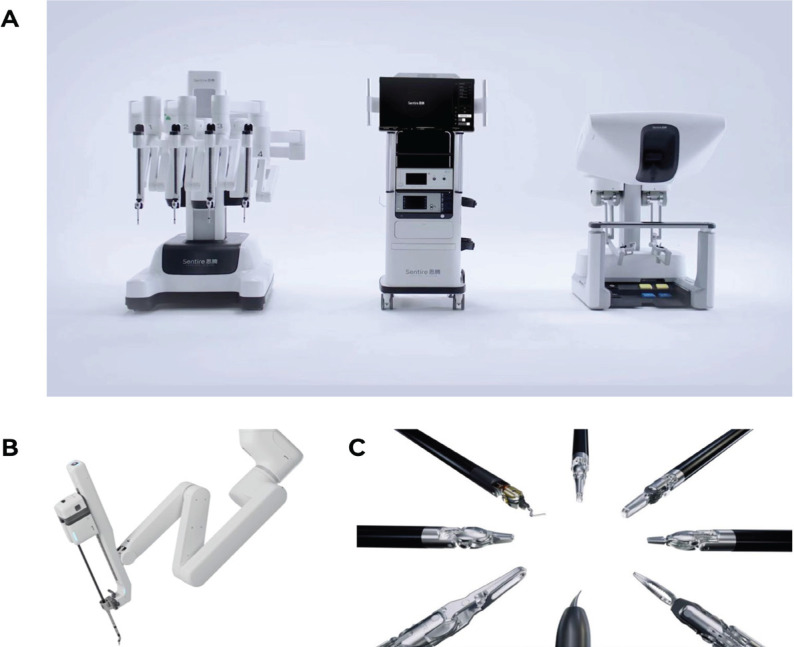
Overview of the Sentire® C1000 Robotic Surgical System Components and Instruments.

The study utilized two different robotic systems for performing RARP, focusing on evaluating their clinical performance and safety. The experimental device used was the Sentire® C1000 Intraperitoneal Laparoscopic Surgical System, developed and manufactured by Shenzhen Cornstone Technology Co., Ltd. The Sentire® C1000 is designed for complex minimally invasive surgeries and comprises three main components: a surgeon console, a patient-side robotic unit, and a vision system (Figure-1A). It is compatible with surgical instruments with an 8mm diameter, featuring a rigid shaft and wristed design. The compatible instruments include a permanent electrocautery hook, monopolar curved scissors, Maryland bipolar forceps, fenestrated bipolar forceps, large needle drivers, extra-large needle drivers, Cadiere forceps, and ExtraGrasp forceps (Figure-1B).

A) The Sentire® C1000 robotic surgical system, designed for complex minimally invasive surgeries, comprises three primary components: the surgeon console, the vision system and the patient-side cart; B) The system supports a range of 8mm diameter surgical instruments with a rigid shaft and wristed design. Compatible instruments include a permanent electrocautery hook, monopolar curved scissors, Maryland bipolar forceps, fenestrated bipolar forceps, large and extra-large needle drivers, Cadiere graspers, and ExtraGrasp forceps.

**Figure 2:**
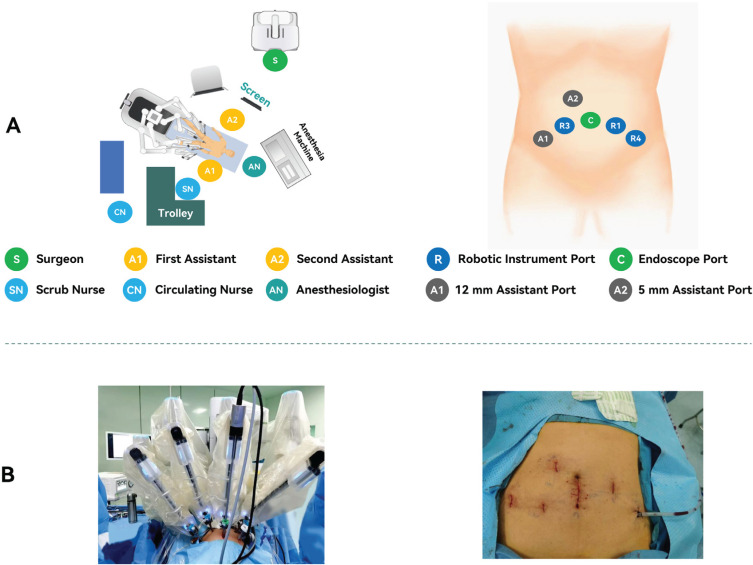
Overview of the Surgical Setup and Trocar Placement.

The surgical setup and trocar placement for RARP performed by the Sentire system are illustrated in Figure-2. For further details, the supplementary video demonstrates the Sentire RARP surgical procedure (Supplementary Video).

This image illustrates the surgical setup and trocar placement in robotic surgery. A) presents a schematic of the operating room layout and the surgical team; B) displays the actual clinical scenario, demonstrating patient positioning, robot placement, and incision sites.

The system in the control group was the da Vinci® SI (IS 3000), which is a well-established robotic system produced by Intuitive Surgical, Inc.

### Outcome Measures

The outcome measures of this study were categorized into preoperative, intraoperative, pathological, and postoperative indicators to comprehensively assess the clinical efficacy and cost-effectiveness of the Sentire® and da Vinci® robotic systems in RARP. Preoperative indicators included patient demographics, comorbidity scores, and disease characteristics. Intraoperative outcomes encompassed surgical approach, operative times, estimated blood loss, intraoperative complications, surgical conversions, transfusion rates, and lymph node dissection. Pathological outcomes involved assessments of pathological grade groups, prostate volume, pathological staging, extraprostatic extension, and surgical margins. Postoperative and follow-up indicators focused on hospital stay duration, readmission rates, PSA recurrence, postoperative PSA levels, and urinary continence at various follow-up intervals. These outcome measures provided a comprehensive evaluation framework to compare the performance of the two robotic systems.

To evaluate the presence of learning curves within the three groups, we analyzed the relationship between the sequence of procedures (time order) and the performance metrics (surgical time and operation time). The learning curve was assessed by examining whether there was a significant reduction in surgical time or operation time as the sequence of procedures progressed within each group.

The *Surgeon Satisfaction Survey* is designed to assess the experience and satisfaction of researchers using an experimental device for urological surgeries. Developed with reference to the Global Evaluative Assessment of Robotic Skills (GEARS) and the Likert scale, the questionnaire is tailored to incorporate the specific performance characteristics of the experimental product. The survey is divided into two sections: System Performance-Related Ratings (Questions 1 to 10) and Surgeon Comfort and Satisfaction Ratings (Questions 11 to 20), each comprising 10 questions. Each question is rated on a scale from 1 to 5, where 1 indicates "Strongly Agree," 2 indicates "Agree," 3 indicates "Neutral," 4 indicates "Disagree," and 5 indicates "Strongly Disagree." A lower score reflects better experience and higher satisfaction with the device during urological surgery (Supplementary Table-1).

**Supplementary Table 1:** Surgeon Satisfaction Questionnaire.

No.	Question
**Part 1: System Performance Evaluation (Questions 1-10, total score: 50)**	
1	The surgical system has a reasonable installation design (consult nursing staff).
2	The surgical system operates smoothly during the procedure.
3	The surgical system runs stably during the procedure.
4	The stereoscopic display on the surgeon console provides clear vision.
5	The stereoscopic display on the surgeon console provides smooth vision.
6	The surgical system enables precise operations in confined spaces.
7	The master-slave control of the surgical system is accurate and responsive during the procedure.
8	The instruments of the surgical system have flexible end-effector manipulation.
9	The instruments of the surgical system provide appropriate grasping force.
10	The energy instruments of the surgical system meet clinical needs for cutting and hemostasis.
**Part 2: Surgeon Comfort and Satisfaction Evaluation (Questions 11-20, total score: 50)**	
11	Using the surgical system did not significantly increase neck muscle fatigue.
12	Using the surgical system did not significantly increase shoulder muscle fatigue.
13	Using the surgical system did not significantly increase arm muscle fatigue.
14	Using the surgical system did not significantly increase wrist muscle fatigue.
15	Using the surgical system did not significantly increase eye fatigue.
16	Using the surgical system did not significantly increase mental stress.
17	The surgical system provides better fluency and comfort compared to traditional laparoscopy.
18	The safety and reliability of the surgical system meet clinical needs during the procedure.
19	The surgical system meets the clinical needs for completing this type of surgery.
20	The surgical system meets clinical needs in terms of interactive experience and control.

The questionnaire consists of two parts: system performance evaluation (Questions 1-10, 10 items) and surgeon comfort and satisfaction evaluation (Questions 11-20, 10 items). Scoring criteria: Each question is scored from 1 to 5, with 1 being "Strongly Agree" and 5 being "Strongly Disagree." Lower scores indicate better experience and satisfaction with the trial device.

The cost evaluation was categorized into direct and indirect costs. Direct costs included surgical fees (which encompass anesthesia fees) and consumable costs, while indirect costs comprised medication expenses, examination fees, and other hospitalization-related expenses (such as laboratory tests, bed charges, nursing care, meals, etc.). All costs were converted to U.S. dollars adjusted for purchasing power parity (PPP$) to enable a standardized and meaningful comparison of the economic impact and cost-effectiveness between the Sentire® and da Vinci® robotic systems in clinical practice. The PPP conversion rate of 3.64 RMB per USD was based on the latest data provided by the World Bank (13).

## Statistical Analysis

Continuous variables were expressed as medians with interquartile ranges (IQRs) to account for non-normal distributions and were compared between the two groups using the Mann-Whitney U test, a non-parametric test suitable for comparing two independent samples. Categorical variables were presented as frequencies and percentages, reflecting their distribution within each group. These categorical variables were analyzed using either the Chi-square test or Fisher's exact test, depending on the expected frequencies; Fisher's exact test was used when the sample sizes were small, or the data were sparse. A two-tailed p-value of less than 0.05 was considered statistically significant, indicating a meaningful difference between the groups. We used the Pearson correlation coefficient (r) to assess the linear relationship between the sequence of procedures and surgical/operation times, with significance tested via a t-test (p < 0.05).

To minimize selection bias and account for baseline differences between the Sentire® and control groups, propensity score matching (PSM) was utilized. Propensity scores were calculated using a logistic regression model that included the following covariates: age, body mass index (BMI), prostate-specific antigen (PSA), prostate volume, pathological ISUP grade, and Charlson comorbidity index (CCI). Each patient in the Sentire® group was matched to up to three patients in the control group using a 1:3 nearest neighbor matching algorithm without replacement, with a caliper width of 0.2 standard deviations of the logit of the propensity score to ensure close matches. This approach minimized baseline differences and improved the comparability between groups, allowing for a more reliable evaluation of the outcomes.

All statistical analyses were conducted using SPSS (Statistical Package for the Social Sciences) software, version 26.0, ensuring rigorous and standardized data processing and interpretation throughout the study.

### Ethical Considerations

The study adhered to the principles outlined in the Declaration of Helsinki and complied with relevant regulations for clinical trials involving medical devices. Informed consent forms were prepared according to these guidelines (14). Prior to enrollment in the study, investigators were required to provide detailed information about the clinical trial to the participants and their families, including the purpose, expected outcomes, potential adverse events, and management strategies. Only after participants fully understood the trial and signed the informed consent form would they be included in the study.

## RESULTS

### Baseline Characteristics

Initially, the baseline characteristics of the Sentire® (experimental) and da Vinci® (control) groups exhibited notable differences in certain clinical parameters. Specifically, the age and PSA levels showed statistically significant disparities before PSM. However, after implementing PSM to balance the cohorts, the distinctions in PSA levels, as well as other variables such as age, BMI, and ISUP grade groups, were effectively neutralized, resulting in no statistically significant differences (all p-values > 0.05). The distributions of age-adjusted CCI and ASA scores, which initially suggested potential variances between groups, were also found to be statistically comparable post-matching. These detailed results are presented in Table-1.

**Table 1:** Baseline Characteristics of RARP Patients treated with Sentire vs. da Vinci Systems Before and After PSM (1:3).

Parameter		Sentire (n=22) Before PSM	Control (n=287) Before PSM	P	Sentire (n=22) After PSM	Control (n=66) After PSM	P
Age, median (IQR)		63.5 (61.0-69.5)	68.0 (62.2-72.5)	**0.034**	63.5 (61.0-69.5)	66.5 (54.0-79.0)	0.215
BMI, median (IQR)		24.3 (22.8-26.9)	25.0 (23.5-27.0)	0.378	24.3 (22.8-26.9)	24.2 (18.3-29.8)	0.837
PSA, median (IQR)		9.9 (6.6-17.5)	6.9 (4.1-14.8)	**0.010**	9.9 (6.6-17.5)	8.8 (5.4-22.3)	0.319
Prostate volume		35.0 (29.3-42.3)	34.1 (27.9-46.8)	0.673	35.0 (29.3-42.3)	34.7 (28.5-46.8)	0.802
**ISUP**				0.087			
	Grade Group 1	6	75		6	18	0.227
	Grade Group 2-3	11	187		11	42	
	Grade Group 4-5	5	25		5	6	
**ASA (n)**				**<0.001**			0.052
	2	21	123		21	48	
	3	1	164		1	18	
**Age-adjust CCI**				**0.049**			0.311
	Low-risk ≤2	7	54		7	17	
	Moderate-risk 3-4	13	138		13	33	
	High-risk ≥5	2	95		2	16	

RARP = robot-assisted radical prostatectomy; PSM = Propensity Score Matching; IQR = Interquartile Range; BMI = Body Mass Index; PSA: Prostate-Specific Antigen; ISUP = International Society of Urological Pathology; ASA = American Society of Anesthesiologists; CCI = Charlson Comorbidity Index

### Surgical Outcomes

Table-2 summarizes the surgical outcomes for both groups. The transperitoneal approach was used by 93.3% of patients in both groups, showing no significant difference (p = 1.000). The median operative time was longer in the Sentire® group (143 minutes, IQR: 115 - 180) compared to the control group (112 minutes, IQR: 100 - 133), with a statistically significant difference (p = 0.024). Console time (p = 0.323), docking time (p = 0.279), and estimated blood loss (p = 0.093) were comparable between groups. No intraoperative complications, surgical conversions, or transfusions occurred in either group. Lymph node dissection was performed in 13.6% of the Sentire® group versus 21.2% of the control group, with no significant difference (p = 0.640).

**Table 2:** Perioperative Outcomes of the Patients in the Sentire and da Vinci group.

	Parametert	Sentire (n=22)	Control (n=66)	P
**Surgical Outcomes**	Transperitoneal Approach, n (%)	14 (93.3)	60 (90.9)	1.000
	Median Operative Time, min	143 (115 – 180)	112 (100 - 133)	**0.024**
	Median Console Time, min	85 (70 −108)	76 (61 - 95)	0.323
	Median Docking Time, min	9.0 (7.0 - 10.5)	6.0 (4.5 - 14.5)	0.279
	Median EBL, mL	50 (30 - 100)	50 (50 - 150)	0.093
	Intraoperative Complications, n (%)	0 (0)	0 (0)	-
	Surgical Conversion, n (%)	0 (0)	0 (0)	-
	Transfusion, n (%)	0 (0)	0 (0)	-
	LND, n (%)	3 (13.6)	14 (21.2)	0.640
**Pathological Outcomes**	Pathological ISUP, n (%)			0.327
	Grade Group 1	0	6 (9.1)	
	Grade Group 2-3	18 (81.8)	53 (80.3)	
	Grade Group 4-5	4 (18.2)	7 (10.6)	
	Pathological Stage >T_3_	3	3	/
	Positive Surgical Margin, n (%)	5 (22.7)	13 (20.0)	1.000
**Postoperative Outcomes and Follow-up**	Postoperative Hospital Stay	2 (2-3)	3 (3-4)	**0.014**
	Postoperative readmission	0 (0)	0 (0)	
	Follow-up			
	1-mo PSA recurrence, n (%)	1 (4.55)	2 (3.03)	1.000
	Median 1-mo PSA, ng/mL	0.018 (0.010 - 0.023)	0.021 (0.010 - 0.030)	0.315
	3-mo PSA recurrence, n (%)	1 (4.55)	3 (4.55)	1.000
	Median 3-mo PSA, ng/mL	0.015 (0.008 - 0.020)	0.018 (0.009 - 0.025)	0.280
	12-mo PSA recurrence, n (%)	2 (9.09)	4 (6.06)	0.625
	Median 12-mo PSA, ng/mL	0.012 (0.006 - 0.018)	0.014 (0.007 - 0.020)	0.410
	Postoperative Urinary Continence, n (%)			
	At 1-mo	7 (31.8)	26 (39.4)	0.798
	At 3-mo	15 (68.2)	41 (62.1)	0.703
	At 12-mo	21 (95.5)	62 (93.9)	1.000
**Surgeon Satisfaction**	**Total**	**22.0 (22.0-23.5)**	**23.0 (22.0-27.0)**	**0.329**
	System Performance	11.0 (10.0-12.0)	11.0 (10.0-13.5)	0.373
	Comfort Level	12.0 (11.0-12.0)	12.0 (11.0-13.5)	0.367
**Cost-effectiveness**	Total (Original), $	8,367.46 (7,594.0–10,153.5)	9307.15 (8800.1–10847.0)	0.058
	Total (PPP), $	16,299.07 (15,544.19 - 18,288.42)	18,129.51 (17,122.28 – 21179.07)	0.067
	Direct Costs (PPP), $			
	Surgery (Including Anesthesia) (PPP), $	8,750.00 (8,720.0 - 9,880.0)	10,500.00 (10,350.0 – 13,050.0)	**0.021**
	Consumable (PPP), $	2,920.00 (2,750.0 - 3,100.0)	3,320.00 (2,910.0 - 3,520.0)	0.114
	Indirect Fees (PPP), $			
	Medication (PPP), $	1,470.0 (1,390.5 - 1,550.9)	1,390.1 (1,311.0 - 1,506.9)	0.454
	Examination (PPP), $	390.00 (340.0 - 450.0)	385.00 (325.0 – 505.0)	0.866
	Other Expenses (PPP), $	2,100.00 (1,180.0 – 4,880.0)	1,690.00 (1,485.0 – 3,210.0)	0.683

### Pathological Outcomes

The pathological outcomes for patients undergoing RARP with the Sentire® (experimental) and da Vinci® (control) systems were compared based on several key parameters (Table-3). The distribution of pathological ISUP Grade Groups showed no significant difference between the two groups (p = 0.327). In the Sentire® group, 81.8% (18 patients) were classified as Grade Groups 2-3, and 18.2% (4 patients) as Grade Groups 4-5. In the control group, 9.1% (6 patients) were in Grade Group 1, 80.3% (53 patients) in Grade Groups 2-3, and 10.6% (7 patients) in Grade Groups 4-5. The median prostate volume was 35.0 cm³ (IQR: 31.1 - 40.3) for the Sentire® group and 34.3 cm3 (IQR: 27.7 - 49.4) for the control group, with no statistically significant difference between them (p = 0.856). Both groups had the same number of patients with a pathological stage greater than T3, with 3 patients in each group. Regarding positive surgical margin (PSM) rates, 22.7% (5 patients) in the Sentire® group and 20.0% (13 patients) in the control group had a positive surgical margin, indicating no significant difference (p = 1.000). Although not statistically significant, there was a slightly higher percentage of patients with positive surgical margins in the Sentire® group compared to the control group.

### Postoperative Outcomes and Follow-up

The median postoperative hospital stay was shorter in the Sentire® group, with a median of 2 days (IQR: 2.0 - 3.0), compared to 3 days (IQR: 3.0 - 4.0) in the control group, showing a statistically significant difference (p = 0.014). There were no readmissions in either group postoperatively (0% for both). During the 12-month follow-up period, PSA recurrence was observed in 2 patients (9.09%) in the Sentire® group and 4 patients (6.06%) in the da Vinci® group, with no statistically significant difference between the two groups (p = 0.625). The median 12-month PSA levels were 0.012 ng/mL (interquartile range [IQR]: 0.006 - 0.018) in the Sentire® group and 0.014 ng/mL (IQR: 0.007 - 0.020) in the da Vinci® group, showing no significant difference (p = 0.410). Urinary continence recovery showed no significant differences between the groups at all follow-up time points. At 1, 3, and 12 months, the continence rates in the Sentire® group were 31.8%, 68.2%, and 95.5%, respectively, compared to 39.4%, 62.1%, and 93.9% in the control group, with p-values of 0.798, 0.703, and 1.000, respectively. All detailed data are listed in Table-2.

### Learning Curve

The analysis of learning curves among the three surgeons revealed no significant evidence of improved efficiency over time for either operative time or console time (Table-3). For Surgeon 1, the Pearson correlation coefficients were 0.16 for operative time and 0.44 for console time, indicating weak to moderate positive correlations, suggesting no learning curve. For Surgeon 2, the coefficients were −0.10 for operative time and 0.07 for console time, reflecting weak negative and near-zero correlations, respectively, with no clear learning trend. Surgeon 3 showed moderate positive correlations, with values of 0.50 for operative time and 0.62 for console time. All p-values were greater than 0.05, indicating no statistical significance.

### Surgeon Satisfaction

The total satisfaction score was similar between the two groups, with a median score of 22.0 (IQR: 22.0 - 23.5) for the Sentire® group and 23.0 (IQR: 22.0 - 27.0) for the control group (p = 0.329). The median score for system performance was 11.0 (IQR: 10.0 - 12.0) in the Sentire® group and 11.0 (IQR: 10.0 - 13.5) in the control group (p = 0.709). For comfort level, the Sentire® group had a median score of 12.0 (IQR: 11.0 - 12.0), while the control group had a slightly higher median score of 13.0 (IQR: 11.5 - 14.0), with a trend towards a difference (p = 0.099).

### Cost-effectiveness

The total costs in Chinese Yuan (RMB) showed a median of ¥60,244.75 (IQR: ¥54,710.21 - ¥73,104.53) for the Sentire® group and ¥66,611.40 (IQR: ¥63,360.72 - ¥77,698.50) for the control group. When converted to U.S. dollars using the updated market exchange rate of 7.2, the Sentire® group had a median of $8,367.46 (IQR: $7,594.00 - $10,153.50), while the control group had a median of $9,307.15 (IQR: $8,800.10 - $10,847.00). Although there was a trend toward cost reduction in the Sentire® group, the difference was not statistically significant (p = 0.058).

Following this, all costs were further adjusted to U.S. dollars using purchasing power parity (PPP$) for standardized comparison. The Sentire® group exhibited lower total costs, with a median of $16,299.07 (IQR: $15,544.19 - $18,288.42), compared to the control group's $18,129.51 (IQR: $17,122.28 - $21,179.07), though this difference did not reach statistical significance (p = 0.067).

In terms of direct costs, a significant reduction was observed in surgery (including anesthesia) expenses, where the Sentire® group had a median cost of $8,750.00 (IQR: $8,720.00 - $9,880.00), notably lower than the control group's $10,500.00 (IQR: $10,350.00 - $13,050.00) (p = 0.021). Consumable costs were also lower in the Sentire® group at $2,920.00 (IQR: $2,750.00 - $3,100.00), compared to $3,320.00 (IQR: $2,910.00 - $3,520.00) in the control group, but this difference was not statistically significant (p = 0.114).

**Table 3:** Perioperative Outcomes of the Patients in the Sentire and da Vinci group.

	Parametert	Sentire (n=22)	Control (n=66)	P
**Surgical Outcomes**	Transperitoneal Approach, n (%)	14 (93.3)	60 (90.9)	1.000
	Median Operative Time, min	143 (115 – 180)	112 (100 - 133)	**0.024**
	Median Console Time, min	85 (70 −108)	76 (61 - 95)	0.323
	Median Docking Time, min	9.0 (7.0 - 10.5)	6.0 (4.5 - 14.5)	0.279
	Median EBL, mL	50 (30 - 100)	50 (50 - 150)	0.093
	Intraoperative Complications, n (%)	0 (0)	0 (0)	-
	Surgical Conversion, n (%)	0 (0)	0 (0)	-
	Transfusion, n (%)	0 (0)	0 (0)	-
	LND, n (%)	3 (13.6)	14 (21.2)	0.640
**Pathological Outcomes**	Pathological ISUP, n (%)			0.327
	Grade Group 1	0	6 (9.1)	
	Grade Group 2-3	18 (81.8)	53 (80.3)	
	Grade Group 4-5	4 (18.2)	7 (10.6)	
	Pathological Stage >T_3_	3	3	/
	Positive Surgical Margin, n (%)	5 (22.7)	13 (20.0)	1.000
**Postoperative Outcomes and Follow-up**	Postoperative Hospital Stay	2 (2-3)	3 (3-4)	**0.014**
	Postoperative readmission	0 (0)	0 (0)	
	Follow-up			
	1-mo PSA recurrence, n (%)	1 (4.55)	2 (3.03)	1.000
	Median 1-mo PSA, ng/mL	0.018 (0.010 - 0.023)	0.021 (0.010 - 0.030)	0.315
	3-mo PSA recurrence, n (%)	1 (4.55)	3 (4.55)	1.000
	Median 3-mo PSA, ng/mL	0.015 (0.008 - 0.020)	0.018 (0.009 - 0.025)	0.280
	12-mo PSA recurrence, n (%)	2 (9.09)	4 (6.06)	0.625
	Median 12-mo PSA, ng/mL	0.012 (0.006 - 0.018)	0.014 (0.007 - 0.020)	0.410
	Postoperative Urinary Continence, n (%)			
	At 1-mo	7 (31.8)	26 (39.4)	0.798
	At 3-mo	15 (68.2)	41 (62.1)	0.703
	At 12-mo	21 (95.5)	62 (93.9)	1.000

Regarding indirect costs, no significant differences were found in medication expenses ($1,470.00 vs. $1,390.10, p = 0.454) or examination fees ($390.00 vs. $385.00, p = 0.866). Similarly, other hospitalization-related expenses were comparable between the groups, with the Sentire® group incurring a median cost of $2,100.00 (IQR: $1,180.00 - $4,880.00), slightly higher than the control group's $1,690.00 (IQR: $1,485.00 - $3,210.00) (p = 0.683).

## DISCUSSION

This study is the first research to report on the clinical use of the Cornerstone Sentire® robotic system for RARP in urology, demonstrating that Sentire® is non-inferior to the da Vinci® system across various surgical and pathological outcomes. As the first study to evaluate the clinical applicability of the Cornerstone Sentire® system, these findings suggest that this new robotic platform can be effectively integrated into clinical practice for RARP, providing a promising new option for urologists.

Robotic systems extend the surgeon's ability to perform delicate operations with increased steadiness and reduced fatigue, leading to improved patient outcomes, shorter recovery times, and fewer complications. Today, the landscape of surgical robotics is rapidly expanding with the introduction of various systems designed to meet diverse surgical needs. Recent innovations such as Hugo® by Medtronic, SHURUI® by Beijing Surgerii, Kangduo® by Sagebot etc., have diversified the options available, each offering unique technological advancements and broadening the scope of robotic applications (15–19).

In comparison to various clinically established robotic surgical systems, the Sentire® system has demonstrated favorable surgical outcomes. All surgeries were completed successfully without any complications, transfusions, or conversions to alternative surgical approaches. The median EBL was 100 mL for both groups, which was comparable to previously published studies (20). The difference in median operative times between the Sentire® and da Vinci® groups—143 minutes (IQR 115 - 180) for Sentire® versus 112 minutes (IQR 100 - 133) for da Vinci® ®—suggests a notable difference; however, the similar console times of 85 minutes (IQR 70 - 108) for Sentire® and 76 minutes (IQR 61 - 95) for da Vinci® indicate that the longer operative time in the Sentire® group may primarily be due to the surgical team's need to adapt to the new system and refine their coordination. This is a common finding in the introduction of new surgical technologies, where overcoming the learning curve is crucial for reducing operative time, as demonstrated in previous studies on robotic surgery systems (21). Notably, the consistent console times across cases suggest that the three surgeons were able to quickly adapt to the Sentire® system with minimal learning curve, demonstrating its user-friendly design and intuitive operation. Additionally, the comparable docking times of 9.0 minutes (IQR 7.0 - 10.5) for Sentire® and 6.0 minutes (IQR 4.5 - 14.5) for da Vinci® ®. Lei et al. reported a docking time of 12.7 minutes for the da Vinci® Si system in prostatectomy, while Menendez reported docking times of 10.45 minutes for the da Vinci® and 18.62 minutes for the Hugo® system (22, 23). Compared to these results, the shorter docking times observed in our study suggest that both the Sentire® and da Vinci® systems are user-friendly and well-designed. The Sentire® system's smaller footprint and increased spatial efficiency allow for better placement and mobility, enhancing its positional flexibility in the operating room. This design advantage is reflected in the docking times, as the system can be positioned more easily and quickly compared to larger systems. These features make Sentire® highly adaptable to different surgical setups, facilitating smoother transitions and potentially reducing overall procedural times, which is particularly beneficial in complex surgical environments like those needed for radical prostatectomies.

The PSM rates observed in our study were 22.7% in the Sentire® group and 20.0% in the control group. Notably, these positive margins were primarily concentrated at the apex of the prostate. In a multi-center study involving 74 surgeons across 10 institutions with a total of 13,090 patients, Bravi et al. found that the likelihood of PSMs in robot-assisted radical prostatectomy decreases significantly as a surgeon's experience increases, dropping from 26% after 10 procedures to 14% after 2,000 procedures (24). All surgeons in our study were experienced robotic specialists, making these PSM rates acceptable given the new robotic system. Additionally, during 1-year follow-up, PSA recurrence was observed in 2 patients in the Sentire® group (9.09%) and 4 patients in the control group (6.06%), who subsequently received adjuvant therapy. A multicenter study reported that BCR-free survival should be around 82.0%-84.8%, indicating satisfactory oncological outcomes in our study, though longer follow-up is needed (25). In our study, urinary continence outcomes were generally comparable to those reported in studies utilizing different robotic systems, achieving a satisfactory level of recovery (26–28). Both the Sentire® and da Vinci® groups showed similar continence rates at 1, 3, and 12 months, with no significant differences between the groups. Given the relatively small sample size, further validation in larger-scale studies is needed to confirm these findings and ensure the robustness of the observed outcomes. Future research will aim to rigorously evaluate urinary continence recovery across different robotic platforms with a more extensive patient cohort.

The median prostate volumes were 35.0 cm³ for the Sentire® group and 34.7 cm³ for the control group, indicating that the study primarily included patients with early-stage prostate cancer, reflected by the low proportion of pT3 cases and ISUP grade 2-3. The low rate of LND further supports this. In the Sentire® group, 3 patients underwent LND without significantly increased console times (92, 124, and 126 minutes), demonstrating that the Sentire® system is capable of performing LND and has potential for routine use in high-risk prostate cancer cases.

The Sentire® robotic system offers a larger primary hand observation field with enhanced imaging and ergonomic design optimized for Asian body types, providing dynamic adjustments for comfort and reducing fatigue during surgery. Features such as "Micro-Damping" and "Active Tremor Filtering" improve operational stability by minimizing hand inertia and filtering tremors. These advantages are reflected in the trend of higher surgeon satisfaction scores for system performance and especially comfort level in the Sentire® group, even though the differences were not statistically significant.

The Sentire® system offers several cost-saving advantages. First, surgery costs (including anesthesia) are significantly lower, largely due to reduced startup costs. Second, consumable costs are also reduced, as the robotic arms can be used up to 12 times, significantly lowering the overall cost per procedure. Finally, the shorter hospital stays associated with the Sentire® system provide additional benefits, such as increased bed turnover and reduced patient burden. However, these advantages did not fully translate into lower indirect costs, likely due to the relatively low cost of hospital beds in China and the more detailed post-surgical monitoring performed in the Sentire® group. The reduction in robotic surgery costs, as demonstrated by the Sentire® system, offers significant advantages for the broader adoption of robotic-assisted surgeries. Lower equipment and consumable costs, along with reduced hospital stays, make robotic systems more accessible and cost-effective for healthcare providers. This cost reduction could facilitate wider adoption of robotic technology in hospitals, not only improving surgical outcomes but also optimizing resource allocation (29).

This study has several limitations. Despite employing the PSM method to reduce potential biases, several constraints remain. First, as an early-stage study, the sample size is small, necessitating future multi-center, large-scale randomized controlled trials (RCTs) to validate the performance of both systems. Second, the study did not utilize the da Vinci® Xi system due to logistical constraints; however, our center has recently introduced it, enabling future RCTs with its inclusion. Third, the surgical scope was limited, and future studies should encompass a broader range of urological procedures, particularly those involving large-volume or locally advanced prostate cancer and complex partial nephrectomies, to better evaluate Sentire®'s applicability, effectiveness, and safety.

## CONCLUSION

In conclusion, the Sentire® system demonstrated non-inferior clinical outcomes to the da Vinci® system in RARP, with the added advantage of cost-effectiveness. Sentire®'s lower equipment and consumable costs make it a compelling option for robotic-assisted surgeries. Moving forward, further studies, including larger scale randomized controlled trials and diverse urological procedures, will be essential to establish Sentire®'s long-term applicability, safety, and effectiveness, potentially expanding its use in various complex urological surgeries.
